# Constituents of the Roots and Leaves of *Ekebergia capensis* and Their Potential Antiplasmodial and Cytotoxic Activities

**DOI:** 10.3390/molecules190914235

**Published:** 2014-09-10

**Authors:** Beatrice N. Irungu, Jennifer A. Orwa, Amra Gruhonjic, Paul A. Fitzpatrick, Göran Landberg, Francis Kimani, Jacob Midiwo, Máté Erdélyi, Abiy Yenesew

**Affiliations:** 1 Kenya Medical Research Institute, Centre for Traditional Medicine and Drug Research, P.O. Box 54840-00200, Nairobi 00200, Kenya; E-Mails: BIrungu@kemri.org (B.N.I.); JOrwa@kemri.org (J.A.O.); 2 Department of Chemistry, University of Nairobi, P.O. Box 30197-00100, Nairobi 00100, Kenya; E-Mail: jmidiwo@uonbi.ac.ke; 3 Sahlgrenska Cancer Centre, University of Gothenburg, Gothenburg SE-405 30, Sweden; E-Mails: amragruhonjic@hotmail.com (A.G.); paul.fitzpatrick@gu.se (P.A.F.); goran.landberg@gu.se (G.L.); 4 Kenya Medical Research Institute, Centre for Biotechnology Research and Development, P.O. Box 54840-00200, Nairobi 00200, Kenya; E-Mail: FKimani@kemri.org; 5 Department of Chemistry and Molecular Biology, University of Gothenburg, Gothenburg SE-412 96, Sweden; 6 Swedish-NMR Centre, University of Gothenburg, Gothenburg SE-405 30, Sweden

**Keywords:** *Ekebergia capensis*, tritepenoid, antiplasmodial, cytotoxicity, Vero, 4T1, HEp2, MDA-MB-231, 3-oxo-12β-hydroxy-oleanan-28,13β-olide

## Abstract

A new triterpenoid, 3-oxo-12β-hydroxy-oleanan-28,13β-olide (**1**), and six known triterpenoids **2**–**7** were isolated from the root bark of *Ekebergia capensis*, an African medicinal plant. A limonoid **8** and two glycoflavonoids **9**–**10** were found in its leaves. The metabolites were identified by NMR and MS analyses, and their cytotoxicity was evaluated against the mammalian African monkey kidney (vero), mouse breast cancer (4T1), human larynx carcinoma (HEp2) and human breast cancer (MDA-MB-231) cell lines. Out of the isolates, oleanonic acid (**2**) showed the highest cytotoxicity, *i.e.*, IC_50_’s of 1.4 and 13.3 µM against the HEp2 and 4T1 cells, respectively. Motivated by the higher cytotoxicity of the crude bark extract as compared to the isolates, the interactions of oleanonic acid (**2**) with five triterpenoids **3**–**7** were evaluated on vero cells. In an antiplasmodial assay, seven of the metabolites were observed to possess moderate activity against the D6 and W2 strains of *P. falciparum* (IC_50_ 27.1–97.1 µM), however with a low selectivity index (IC_50_(vero)/IC_50_(*P. falciparum-D6*) < 10). The observed moderate antiplasmodial activity may be due to general cytotoxicity of the isolated triterpenoids.

## 1. Introduction

*Ekebergia capensis* Sparrm (Meliaceae) is a deciduous tree attaining a height of up to 30 m. It is widely distributed in the central and Nyanza regions of Kenya [1,2], and is also widespread in South Africa, Swaziland, Zimbabwe, Uganda and Ethiopia. The Zulu community in South Africa uses its wood to facilitate childbirth [3]. In Kenya, the Sabaot community uses its leaf macerations internally and externally to treat headache, fever, cough and skin diseases, while the Agĩkũyũ community treats diarrhea with its stem bark [1,4]. Pharmacological studies have indicated antiplasmodial, antiinflammatory, hypotensive, uterotonic and antituberculotic activities of the crude extracts of this plant [3,5,6,7,8], providing scientific support for their indigenous use. Phytochemical investigations of its stem bark led to isolation of triterpenoids, steroids and flavonoids [3,9,10]. The safe application of *E. capensis* in traditional medicine requires the presence of metabolites with useful pharmacological properties and low toxicity levels. Here, the isolation, spectroscopic identification, and biological evaluation of the constituents of *E. capensis* root bark and leaf extracts are reported, with special attention given to the evaluation of the cytotoxicity of the constituents.

## 2. Results and Discussion

### 2.1. Isolation and Spectroscopic Identification

**Figure 1:**
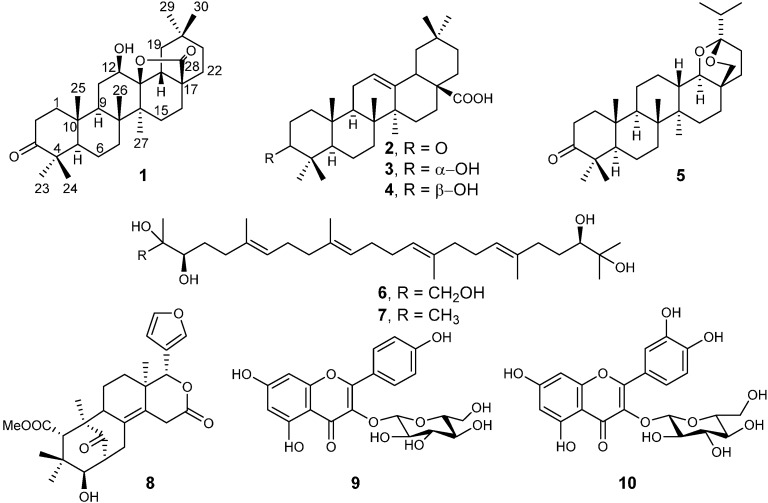
Compounds **1**–**10** isolated from *Ekebergia capensis*.

The air-dried root bark and the leaves of *E. capensis* were extracted separately with MeOH-CH_2_Cl_2_ (1:1) at room temperature. The two extracts were subjected to column chromatography on silica gel yielding ten metabolites (Figure 1), which were characterized by NMR and MS. Out of the seven constituents isolated from the roots, one (compound **1**) was new, whereas six were known triterpenoids, namely oleanonic acid (**2**) [3,9], 3-*epi*-oleanolic acid (**3**) [3,9], oleanolic acid (**4**) [9], ekeberin A (**5**) [9], 2-hydroxymethyl-2,3,22,23-tetrahydroxy-6,10,15,19,23-pentamethyl-6,10,14,18-tetracosatetraene (**6**) [9,10], and 2,3,22,23-tetrahydroxy-2,6,10,15,19,23-hexamethyl-6,10,14,18-tetracosatetraene (**7**) [9,10]. From the leaves, proceranolide (**8**) [9], kaempferol-3-*O-*β-d-glucopyranoside (**9**) [11], and quercetin-3-*O*-β-d-glucopyranoside (**10**) [12] were identified.

**Figure 2:**
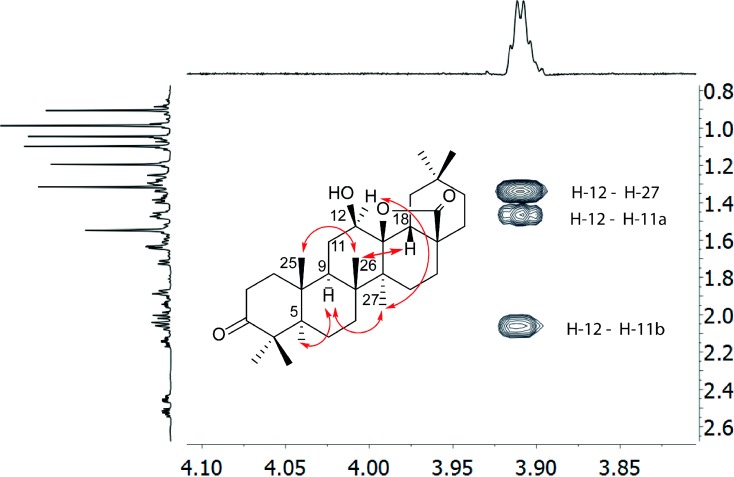
Key NOE correlations observed for compound **1** (mixing time 700 ms, CDCl_3_, 25 °C, 799.88 MHz), allowing determination of its relative configuration, are shown to the left. An expansion of the NOESY spectrum showing the characteristic NOE correlations of H-12 is shown to the right. The NOE correlation of H-12 and H-27, and the absence of NOE between CH-12 and H-26 indicate the β-orientation of OH-12. The full NOESY spectrum is shown in the Supporting Information.

Compound **1** was isolated as a white amorphous powder. Its HR(ESI)MS analysis suggested the molecular formula C_30_H_46_O_4_ (observed [M+H]^+^, *m/z* 471.3386, calcd. 471.3474). The presence of seven methyl singlets in its ^1^H-NMR spectrum at δ_H_ 1.32, 1.20, 1.10, 1.05, 0.91 integrating for 3H each, a methyl at δ_H_ 0.99 integrating for 6H (Table 1), and that of thirty ^13^C-NMR signals including an oxygenated methine (δ_C_ 76.2), a quaternary carbon (δ_C_ 90.6), and two carbonyls (δ_C_ 217.7 and 179.9) was compatible with a pentacyclic triterpenoid skeleton. This presumption was supported by the high similarity of its NMR data to that of the oleanane triterpenoid 3-oxo-11α-12α-epoxy-oleanan-28,13β-olide, previously isolated from *Cedrela montana* [13]. The HMBC correlations (Table 1) of the carbonyl carbon at δ_C_ 217.7 ppm to the methyl protons at δ_H_ 1.10 ppm (H-23) and δ_H_ 1.05 ppm (H-24), and to the methylene protons at δ_H_ 2.53 and 2.45 ppm (CH_2_-2) suggests its C-3 position in the triterpenoid backbone. The placement of a hydroxyl group at C-12 (δ_C_ 76.2 ppm) was established following the ^1^H,^1^H COSY correlation of H-11 and H-12, and the HMBC correlation of H-12 (δ_H_ 3.91 ppm) with C-9 (δ_C_ 44.0 ppm), C-13 (δ_C_ 90.6 ppm) and C-14 (δ_C_ 42.3 ppm). This was further confirmed by the HMBC correlation of H-11 (δ_H_ 2.06, 1.46 ppm) and H-9 (δ_H_ 1.72 ppm) to C-12 (δ_C_ 76.2 ppm). The relative configuration of the C12-hydroxyl group was determined based on the NOE observed between H-12 (δ_H_ 3.91 ppm) and H-27 (δ_H_ 1.32 ppm), revealing their *syn* orientation (Figure 2). This assignment was further supported by the absence of NOE between H-12 and CH_3_-26 (δ_H_ 1.20 ppm). Small, comparable ^3^*J*_H12-H11a_ and ^3^*J*_H12-H11b_ indicate the gauche orientation of H-12 to both H-11a and H-11b, and thus its *pseudo*-equatorial orientation. The ^13^C-NMR shifts δ_C_ 179.9 ppm (C-28) and δ_C_ 90.6 ppm (C-13) are typical of a 28,13β-lactone moiety [13,14,15,16]. It should be noted that so far all naturally occurring triterpenes possessing a 28,13-lactone moiety were reported to have 28,13β-configuration [13,17,18]. The relative orientation of the bridgehead methyl groups and protons was elucidated based on their NOE correlations, shown in Figure 2. Hence, the absence of NOE between H-18 and H-27, H-5 and H-25, and H-26 and H-27 is diagnostic for their *anti-*orientation. The observed NOEs revealed that H-5, H-9, H-12 and H-27 are α-oriented, whilst H-18, H-25, and H-26 are β-oriented. The assigned relative configuration is in excellent agreement with previous literature reports [13,17,18]. On the basis of the above spectroscopic data, the new compound was characterized as 3-oxo-12β-hydroxy-oleanan-28,13β-olide (**1**). Compounds **2**–**10** were identified by their 2D NMR and MS data (see Supporting Information). Their structures were confirmed by comparison of their spectroscopic data to that previously reported [9,10,11,12].

**Table 1:** ^1^H- (799.88 MHz) and ^13^C- (201.20 MHz) NMR data for 3-oxo-12β-hydroxy-oleanan-28,13β-olide (**1**) in CDCl_3_.

	δ_H_ (I, multiplicity, *J* in Hz)	δ_C_	HMBC (^2^*J*, ^3^*J*)
1	1.47 (1H, *ddd*, 7.6, 9.8, 12.5)	39.8	C2, C3, C5, C10, C25
	1.94 (1H, *ddd*, 4.4, 7.6, 12.5)		
2	2.53 (1H, *ddd*, 7.6, 9.8, 15.7)	34.1	C1, C3, C4, C10
	2.45 (1H, *ddd*, 4.4, 7.6, 15.7)		
3	-	217.7	-
4	-	47.5	-
5	1.39 (1H, *dd*, 2.7, 12.0)	55.0	C4, C6, C7, C9, C10, C23, C25
6	1.55 (1H, *m*)	19.2	C7, C8, C10, C26
	1.47 (1H, *m*)		C7, C8, C10, C25, C26
7	1.61 (1H, *m*)	33.5	C5, C6, C8, C26
	1.30 (1H, *m*)		C5, C8, C9, C26
8	-	42.3	-
9	1.72 (1H, *dd*, 2.3, 13.1)	44.0	C1, C5, C8, C10, C11, C25, C26
10	-	36.3	-
11	2.06 (1H, *m*)	29.3	C8, C9, C10, C12, C13
	1.46 (1H, *m*)		
12	3.91 (1H, *dd*, 3.3, 3.3)	76.2	C9, C13, C14
13	-	90.6	-
14	-	42.3	-
15	1.88 (1H, *ddd*, 2.6, 2.6, 12.4)	28.2	C14, C16, C17, C18, C27
	1.20 (1H, *m*)		
16	2.14 (1H, *ddd*, 5.9, 13.3, 13.3)	21.3	C17, C18, C22, C28
	1.29 (1H, *m*)		-
17	-	44.9	-
18	2.05 (1H, *dd*, 3.5, 13.4)	51.3	C12, C13, C14, C16, C19, C20
19	2.01 (1H, *dd*, 13.1, 13.4)	39.7	C17, C18, C20, C21, C29, C30
	1.87 (1H, *dd*, 3.5, 13.1)		
20	-	31.8	-
21	1.38 (1H, *m*)	34.3	C20, C22, C30
	1.27 (1H, *m*)		
22	1.64 (2H, *m*)	27.6	C16, C17, C18, C20, C28, C29
23	1.10 (3H, *s*)	26.7	C3, C4, C5, C24
24	1.05 (3H, *s*)	21.2	C3, C4, C5, C23
25	0.99 (3H, *s*)	16.4	C1, C5, C9, C10
26	1.20 (3H, s)	18.4	C7, C9, C13, C14, C27
27	1.32 (3H, *s*)	18.6	C8, C13, C14, C15
28	-	179.9	-
29	0.99 (3H, *s*)	33.4	C19, C20, C21,C30
30	0.91 (3H, *s*)	24.0	C19, C20, C21,C29

### 2.2. Antimalarial Activity and Cytotoxicity

Previous phytochemical studies have revealed the *in vitro* antiplasmodial potency of some triterpenoids [9,19]. Therefore, the constituents of *E. capensis* were tested against the chloroquine sensitive (D6), and the chloroquine resistant (W2) strains of *Plasmodium falciparum*. Seven metabolites showed moderate *in vitro* antiplasmodial activity against the D6 (IC_50_ = 27–97.1 µM), whilst four had moderate activity against the W2 (IC_50_ = 64–82.7 µM, Table 2) strains. Due to the low amount isolated, the bioactivity of **1** was not evaluated. A moderate *in vitro* antiplasmodial activity of oleanolic acid (**4**) against the 3D7 strain of *P. falciparum* has been previously reported [20].

For evaluation of the possible risks associated with the application of *E. capensis* extracts in traditional medicine, we have studied their cytotoxicity using vero cells (Table 2). The flavonoid containing leaf extract showed a low, whereas the root bark extract, rich in triterpenoids, a high toxicity towards vero cells. The latter observation is in agreement with the cytotoxicity reported for triterpenoids previously isolated from other plants [11,16].

The substantial toxicity of the root bark extract of *E. capensis* towards vero cells motivated us to assess the anticancer properties of its metabolites. Indeed, compounds **2**, **3**, **6** and **7** exhibited even higher toxicity against the 4T1 murine breast cancer cell line than against vero cells (Table 2). This toxicity and the corresponding low selectivity index (IC_50_(vero)/IC_50_(D6) < 10) suggest that the observed moderate antiplasmodial activity of the metabolites likely originates from their cytotoxicity. For further evaluation of anticancer potency, we assessed the toxicity of the samples against the HEp2 and MDA-MB-231 human cancer cell lines (Table 2). Of the seven triterpenoids isolated from the root bark, oleanonic acid (**2**) showed the highest cytotoxicity, 1.4 μM against HEp2 cells. Here, it should be noted that the toxicity of **2** against “normal” vero cells is as low as that of the positive control chloroquine, motivating its further assessment as a possible anticancer agent.

**Table 2:** The antiplasmodial and cytotoxic activities of *Ekebergia capensis* leaf and root bark crude extracts and their constituents.

	IC_50_^a^					
	D6 ^b^	W2 ^b^	Vero	4T1 ^c^	HEp2	MDA-MB-231
**roots**	18.2 ± 0.1	34 ± 0.8	2.8 ± 0.1	9.3 ± 0.1	61 ± 1.4	n.d.
**leaves**	44.9 ± 0.8	45.3 ± 0.5	97.8 ± 0.8	82.1 ± 5.7	71.6 ± 1.8	n.d.
**2**	38.8 ± 0.5	76.7 ± 4.0	35.8 ± 1.3	13.3 ± 0.2	1.4 ± 0.1	>212
**3**	205.0 ± 3.0	179.4 ± 6.0	58.0 ± 5.2	30.3 ± 2.6	29.8 ± 0.3	36.54 ± 0.02
**4**	49.6 ± 2.3	82.7 ± 2.0	112.0 ± 5.1	117.6 ± 2.6	134.9 ± 0.7	39.82 ± 0.03
**5**	182.2 ± 6.0	>219	>219	163.2 ± 4.3	>219	n.d.
**6**	27.1 ± 0.4	66.9 ± 0.6	35.7 ± 2.1	30.2 ± 1.3	38.4 ± 0.8	36.69 ± 0.04
**7**	56.1 ± 0.4	64.3 ± 1.0	24.7 ± 1.8	22.5 ± 3.2	35.5 ± 3.1	> 209
**8**	84.7 ± 0.8	150.2 ± 3.0	>213	>213	>213	n.d.
**9**	97.1 ± 1.0	105.8 ± 0.5	>223	>223	>223	>223
**10**	42.9 ± 0.3	105.8 ± 1.0	>216	>216	>216	>216

^a^ IC_50_: half maximal inhibitory concentration, given in μM for pure compounds and in μg/mL for crude extracts. The mean values of at least three independent experiments are reported; ^b^ Chloroquine was used as positive control (IC_50_(D6) = 7.7 ± 1 nM, IC_50_(vero) = 43.9 ± 0.5 µM); ^c^ Positive control: podophyllum resin, IC_50_(4T1) = 0.47 ± 0.05 µg/mL; n.d., not determined.

It is interesting to note that the root extract was more toxic to vero cells than to HEp2 cells, while the reverse situation (Table 2) was observed for oleanonic acid (**2**). The high toxicity of the root extract to vero cells may be due to synergistic effect of some of its constituents. Therefore, the interaction of oleanonic acid (**2**), the most toxic metabolite against HEp2 cells, with triterpenoids **3**–**7** against vero cells was evaluated. 3-*Epi*-oleanolic acid (**3**) markedly antagonized the cytotoxic effects of **2** at all concentrations tested, whereas its stereoisomer (**4**) showed only slight antagonistic effect (Table 3). The cytotoxicity of oleanonic acid (**2**) was antagonized by high concentrations of ekeberin A (**5**), but at lower relative concentrations **5** enhanced the toxicity of **2**. Triterpenoids **6** and **7** showed weak antagonistic effects. Overall, no significant synergistic effect was observed.

**Table 3:** Interaction of oleanonic acid (**2**) (IC_50_ 14.84 µg/mL) with other constituents (**3**–**7**) of the root bark extract of *Ekebergia capensis* against vero cells.

Compound	IC_50_ (μg/mL)			
	0:1 ^a^	1:3 ^a^	1:1 ^a^	3:1 ^a^
3	22.5 ^b^	9.8 ^c^	5.2 ^c^	5.8 ^c^
4	40.3 ^b^	3.3 ^c^	4.7 ^c^	3.6 ^c^
5	>100 ^b^	<4 ^c^	<1.4 ^c^	<1.3 ^c^
6	13.6 ^b^	2.2 ^c^	2.2 ^c^	2.3 ^c^
7	11 ^b^	2 ^c^	1.9 ^c^	2.2 ^c^

^a^ Ratio of oleanonic acid (**2**) *versus* various constituents of the root extract; ^b^ IC_50_ (µg/mL) in the absence of **2**; ^c^ ∑FIC.

## 3. Experimental Section

### 3.1. General Information

Column chromatography was carried out on silica gel 60, 0.06–0.2 mm, 70–230 mesh ASTM, obtained from Scharlab SL, or on Sephadex^®^ LH-20, purchased from Fluka (Buchs, Switzerland). PTLC was performed on locally made 20 × 20 cm glass plates using Silica gel G/UV 254 (Macherey-Nagel, Düren, Germany). TLC was run using fluorescent silica gel 60 obtained from Fluka, and was visualized under UV light, 254 or 366 nm, followed by spraying with 1% vanillin dissolved in sulphuric acid. LC(ESI)MS spectra was acquired on a PE SCIEX API 150EX instrument (Perkin Elmer, Waltham, MA, USA) equipped with a Turbolon spray ion source (30 eV ionization energy) and a Gemini 5 mm C-18 110 Å HPLC column, using water:acetonitrile gradient (80:20 to 20:80). Preparative HPLC separation was carried out on a Waters 600E HPLC system (Waters Corp, Milford, MA, USA) using the Chromulan software (Pikron Ltd., Praha, Czech Republic) with a Kromasil C-8 250 × 25 mm C-8 column with water-acetonitrile eluent mixtures. For structure elucidation gCOSY [21], gNOESY [22], gHSQC [23] and gHMBC [24] NMR spectra were acquired on an 800 MHz (Bruker BioSpin AG, Fällanden, Switzerland) or on Varian 500 and 400 MHz spectrometers (Agilent, Palo Alto, CA, USA). Spectra were processed using the MestReNova (v9.0.0) software. Chemical shifts were referenced indirectly to tetramethylsilane via the residual solvent signal (CDCl_3_, ^1^H at 7.26 ppm and ^13^C at 77.16 ppm). High resolution mass analysis (Q-TOF-MS) was performed by Stenhagen Analyslab AB (Gothenburg, Sweden) using a Micromass Q-TOF micro instrument (Waters Corp, Milford, MA, USA) equipped with a lockmass-ESI source.

### 3.2. Plant Material

The root bark and leaves of *Ekebergia capensis* were collected from Gakoe forest, Kiambu County, in April, 2013. The plant was authenticated by Mr. Patrick Mutiso and a voucher specimen (BN/1/2013) was deposited at the Herbarium of the School of Biological Sciences, University of Nairobi.

### 3.3. Extraction and Isolation

The air dried and ground root bark (600 g) of *E. capensis* was extracted twice with MeOH–CH_2_Cl_2_ (1:1, 1 L) for 48 h at room temperature. The filtrate was dried *in vacuo* to yield a blackish sticky solid (97 g). A 30 g portion of the extract was fractionated using gradient column chromatography with a petroleum ether (b.p.: 60–80 °C) ethyl acetate gradient in the following ratios: 100:0; 19:1; 9:1; 4:1; 3:2; 1:1; 2:3; 1:4; 0:100. A total of 86 fractions, *ca.* 250 mL each, was collected and combined into 22 fractions (labeled A to V) based on TLC. Fraction B was crystallized in acetone to yield oleanonic acid (**2**, 2.7 g). The supernatant of fraction B, following crystallization, was separated on Sephadex LH-20 with methanol eluent to yield fraction B1, which was crystallized in acetone to yield ekeberin A (**5**, 2.1 mg). Fraction C was precipitated with acetone to yield white solid (762.8 mg) that was further purified on reversed-phase HPLC (CH_3_OH-H_2_O gradient) to yield 3-*epi*-oleanolic acid (**3**, 7.4 mg). Fraction D was separated on PTLC with an 8:2 mixture of petroleum ether and acetone to yield 3-oxo-12α-hydroxy-oleanan-28,13β-olide (**1**, 2.3 mg) as a white, amorphous solid. Fraction G yielded oleanolic acid (**4**, 284 mg) as white powder. Fractions I and M were separated on PTLC with petroleum ether-acetone (7:3) eluent to yield 2-hydroxymethyl-2,3,22,23-tetrahydroxy-6,10,15,19,23-pentamethyl-6,10,14,18-tetracosatetraene (**6**, 30.3 mg) and 2,3,22,23-tetrahydroxy-2,6,10,15,19,23-hexamethyl-6,10,14,18-tetracosate (**7**, 90.6 mg), respectively.

The dried and ground leaf of *E. capensis* (500 g) was extracted with MeOH–CH_2_Cl_2_ (1:1, 1 L) to yield 21 g of a greenish sticky solid. A 20 g portion was fractionated on a silica gel column eluting with a mixture of petroleum ether and acetone with the gradient 100:0, 9.75: 0.25, 9.5:0.5, 9.25:0.75, 9:1, 8.75:1.25, 8.5:1.5, 8.25:1.75, 8:2, 7:3, 1:1, 0:100. A total of 81 fractions, *ca.* 250 ml each, were collected and combined into 22 fractions labeled as A-V, upon TLC analysis. Fraction Q was further fractionated with reverse-phase HPLC using CH_3_OH:H_2_O gradient to yield fractions Q2 and Q6. Fraction Q2 was purified on preparative TLC with *iso*-hexane:acetone (4:1) to yield proceranolide (**8**, 5.7 mg). Fractions T and W were purified on reversed-phase HPLC using a CH_3_OH:H_2_O gradient to yield kaempferol-3-*O*-β-d-glucopyranoside (**9**, 3.5 mg) and quercetin-3-*O*-β-d-glucopyranoside (**10**, 10.1 mg), respectively.

### 3.4. Cytotoxicity Assays

A rapid colorimetric assay was carried out using 3-(4,5-dimethylthiazol-2-yl)-2,5-diphenyltetrazolium bromide (MTT) [25,26]. This assay is based on the ability of a mitochondrial dehydrogenase enzyme from viable cells to cleave the tetrazolium rings of the pale yellow MTT and thereby form dark blue formazan crystals, which are largely impermeable to cell membranes, resulting in their accumulation within healthy cells. The amount of generated formazan is directly proportional to the number of cells [25]. In this assay, the mammalian cell lines African monkey kidney (vero), mouse breast cancer (4T1) and human larynx carcinoma (HEp2) were used. Cells were maintained in Eagle’s Minimum Essential Medium (MEM) containing 10% fetal bovine serum (FBS). A cell density of 20.000 cells per well in 100 μL were seeded on 96-well plates and incubated for 12 h at 37 °C and 5% CO_2_ to attach to the surface. Samples of the tested extracts and isolated compounds were added to the cultured cells in rows H-B over a concentration range of 0.14 to 100 μg/mL, whereas wells 1–8 of row A served as untreated controls and wells 9–12 as blank (1% DMSO, v/v). The plates were incubated for 48 h at 37 °C and 5% CO_2_, followed by an addition of 10 µL MTT viability indicator reagent. The plates were incubated for additional 4 h at the same conditions. Next, all media was removed from the plates and 100 µL DMSO was added to dissolve the formazan crystals. The plates were read on a Multiskan EX Labsystems scanning multi-well spectrophotometer at 562 nm, and 620 nm as reference. The results were recorded as optical density (OD) per well at each drug concentration. The data was transferred into the software Microsoft Excel and expressed as percentage of the untreated controls. Percentage cytotoxicity (PC) as compared to the untreated controls was calculated as PC = [A − B/A] × 100, where A is the mean OD of the untreated cells and B is the mean OD at each drug concentration [26]. The drug concentration required for 50% inhibition of cell growth was estimated using nonlinear regression analysis of the dose- response curve.

Cytotoxicity tests on MDA-MB-231 cells were carried out following a previously described procedure [27]. MDA-MB-231 human breast cancer cells were cultured in Dulbecco’s modified eagle medium (DMEM) supplemented with 10% (v/v) fetal bovine serum, 2 mM L-glutamine, 100 units/mL penicillin and 100 μg/mL streptomycin at 37 °C in humidified 5% CO_2_. For cytotoxicity assays, cells were seeded in 96-well plates at optimal cell density (10,000 cells per well) to ensure exponential growth for the duration of the assay. After a 24 h preincubation growth, the medium was replaced with experimental medium containing the appropriate drug concentrations or vehicle controls (0.1% or 1.0% v/v DMSO). After 72 h of incubation, cell viability was measured using Alamar Blue reagent (Invitrogen Ab, Lidingö, Sweden) according to the manufacturer’s instructions. Absorbance was measured at 570 nm with 600 nm as a reference wavelength. Results were expressed as the mean ± standard error for six replicates as a percentage of vehicle control (taken as 100%). Experiments were performed independently at least six times. Statistical analyses were performed using a two-tailed Student’s *t*-test. *p* < 0.05 was considered to be statistically significant.

The interaction of oleanonic acid (**2**) with other triterpenoids was studied using the fixed concentration ratios oleanonic acid: ‘other triterpenoid’ 0:1, 1:3, 1:1, 3:1, 1:0. The vero cell cytotoxicity assay was used, as described above, to evaluate the toxicity of the mixtures. To determine whether there was synergy, additive effect or antagonism, the sum of fractional inhibition concentration (∑FIC) was calculated using the formula A_x_/A_y_ + B_x_/B_y_ = K, where A_x_ and B_x_ are the IC_50_s when the substances are used in combination, and A_y_ and B_y_ are the IC_50_s, when the substances are used alone. The data was scored with the scale ∑FIC < 1: synergism, 2 > ∑FIC ≥ 1: additive, 4 > ∑FIC ≥ 2: slight antagonism, ∑FIC ≥ 4: marked antagonism [28].

### 3.5. In Vitro Antiplasmodial Assay

Continuous *in vitro* cultures of asexual erythrocytic stages of Indochinese chloroquine-resistant W2 and Sierra Leonean chloroquine-sensitive D6 strains of *P. falciparum* were maintained following the modified procedure described by Trager and Jensen [29]. Drug assay was carried out following a modification of the semiautomated microdilution technique, which measures the ability of the extracts to inhibit the incorporation of (G-^3^H) hypoxanthine into the malaria parasite [30]. Plates were harvested onto glass fibre filters and (G-^3^H) hypoxanthine uptake was determined using a micro-beta trilux liquid scintillation and luminescence counter (Wallac, MicroBeta TriLux) with the results recorded as counts per minute (cpm) per well at each drug concentration. Data was transferred into the software Microsoft Excel and was expressed as the percentage of the untreated controls. The drug concentration required for 50% inhibition of (G-^3^H) hypoxanthine incorporation into parasite nucleic acid was calculated with nonlinear regression analysis of the dose-response curve. The criterion described by Batista and co-workers for scoring activity was adopted [31], *i.e.*, IC_50_ < 1 μM: highly active; 20 μM > IC_50_ ≥ 1: active; 100 μM > IC_50_ ≥ 20: moderately active; IC_50_ > 100 inactive.

## 4. Conclusions

Phytochemical analysis indicated that the root bark of *E. capensis* contains pentacyclic triterpenoids. The major secondary metabolites of the root bark are present in the stem bark as well [9]. The triterpenoid 3-oxo-12β-hydroxy-oleanan-28,13β-olide (**1**) is a new compound. Most constituents of the root bark showed moderate antiplasmodial activity with low selectivity indices, revealing their limited applicability for antimalarial drug development. The triterpenoids **3**–**7** showed comparable cytotoxicity towards “normal” (vero) and tumor cells, whereas oleanonic acid (**2**) possessed low toxicity against vero cells yet high toxicity (1.4 μM) against the 4T1 and HEp2 cancer cell lines. Its low activity against MDA-MB-231 human breast cancer cells indicates some selectivity of its anticancer activity. No significant synergism on the cytotoxicity of oleanonic acid (**2**) with other constituents of the root bark was detected. Based on the above observations we recommend further evaluation of oleanonic acid (**2**) on additional normal and cancerous cell lines for careful evaluation of its potency as anticancer lead. Whereas the root bark extract of *E. capensis* possesses high toxicity against “normal” (2.8 μM, vero) cells, no toxicity for its leaf extract or its constituents was observed. Although *in vitro* toxicity cannot be directly extrapolated to *in vivo* toxicity, our observations suggest a low risk of the indigenous application of *E. capensis* leaf extracts, but a substantial risk associated with the traditional medicinal use of its root extracts.

## Supplementary Materials

Supplementary materials can be accessed at: http://www.mdpi.com/1420-3049/19/9/14235/s1.

## Supplementary Files

Supplementary File 1

## References

[1] Gachathi M. (2007). Kikuyu Botanics Dictionary: A Guide to Plant Names Uses and Culture Values.

[2] Beentje H.J. (1994). Kenya Trees, Shrubs, and Lianas.

[3] Sewram V., Raynor M.W., Mulholland D.A., Raidoo D.M. (2000). The uterotonic activity of compounds isolated from the supercritical fluid extract of *Ekebergia capensis*. J. Pharm. Biomed. Anal..

[4] Okello S.V., Nyunja R.O., Netondo G.W., Onyango J.C. (2010). Ethnobotanical study of medicinal plants used by Sabaots of Mt. Elgon Kenya. Afr. J. Tradit. Complement. Altern. Med..

[5] Kamadyaapa D.R., Gondwe M.M., Moodley K., Ojewole J.A.O., Musabayane C.T. (2009). Cardiovascular effects of Ekebergia capensis Sparrm (Meliaceae) ethanolic leaf extract in experimental animal paradigms. Cardiovasc. J. Afr..

[6] Lall N., Meyer J.J.M. (1999). *In vitro* inhibition of drug-resistant and drug-sensitive strains of Mycobacterium tuberculosis by ethnobotanically selected South African plants. J. Ethnopharmacol..

[7] Mulaudzi R.B., Ndhlala A.R., Kulkarni M.G., Finnie J.F., Staden J.V. (2013). Anti-inflammatory and mutagenic evaluation of medicinal plants used by Venda people against venereal and related diseases. J. Ethnopharmacol..

[8] Muregi F.W., Chhabra S.C., Njagi E.N.M., Lang’at-Thoruwa C.C., Njue W.M., Orago A.S., Omar S.A., Ndiege I.O. (2004). Anti-plasmodial activity of some Kenyan medicinal plant extracts singly and in combination with chloroquine. Phytother. Res..

[9] Murata T., Miyase T., Muregi F.W., Naoshima-Ishibashi Y., Umehara K., Warashina T., Kanou S., Mkoji G.M., Terada M., Ishih A. (2008). Antiplasmodial triterpenoids from *Ekebergia capensis*. J. Nat. Prod..

[10] Nishiyama Y., Moriyasu M., Ichimaru M., Tachibana Y., Kato A., Mathenge S.G., Nganga J.N., Juma F.D. (1996). Acyclic triterpenoids from *Ekebergia capensis*. Phytochemistry.

[11] Kim K.H., Choi S.U., Lee K.R. (2012). Cytotoxic triterpenoids from *Berberis koreana*. Planta Med..

[12] Kim H.Y., Moon B.H., Lee H.J., Choi D.H. (2004). Flavonol glycosides from the leaves of *Eucommia ulmoides O*. with glycation inhibitory activity. J. Ethnopharmacol..

[13] Castellanos L., de Correa R.S., Martínez E., Calderon J.S. (2002). Oleanane triterpenoids from *Cedrela montana* (Meliaceae). Z. Naturforsch. C.

[14] Konoike T., Takahashi K., Araki Y., Horibe I. (1997). Practical partial synthesis of myriceric acid A, and endothelin receptor antagonist, from oleanolic acid. J. Org. Chem..

[15] Salvador J.A.R., Moreira V.M., Pinto R.M.A., Leal A.S., Paixao J.A. (2012). Efficient oxidation of oleanolic acid derivatives using magnesium bis(monoperoxyphtalate) hexahydrate (MMPP): A convenient two-step procedure towards 12-oxo-28-carboxylic acid derivatives. Beilstein J. Org. Chem..

[16] Leal A.S., Wang R., Salvador J.A.R., Jing I. (2013). Synthesis of novel heterocyclic oleanolic acid derivatives with improved antiproliferative activity in solid tumor cells. Org. Biomol. Chem..

[17] Hu J., Wu G., Xu Y., Xiao G., Lei P. (2012). 12α-Hydroxy-3,27-dioxooleanano-28,13-lactone. Acta Crystallogr. Sect. E.

[18] Ikuta A., Tomiyasu H., Morita Y., Yoshimura K. (2003). Ursane- and oleanane-type triterpenes from *Ternstroemia gymnanthera callus* tissues. J. Nat. Prod..

[19] Ngouamegne E.T., Fongang R.S., Ngouela S., Boyom F.F., Rohmer M., Tsamo E., Gut J., Rosenthal P.J. (2008). Endodesmiadiol, a friedelane triterpenoid, and other antiplasmodial compounds from *Endodesmia calophylloides*. Chem. Pharm. Bull..

[20] Sairafianpour M., Bahreininejad B., Witt M., Ziegler H.L., Jaroszewski J.W., Staerk D. (2003). Terpenoids of *Salvia hydrangea*: Two new, rearranged 20-norabietanes and the effect of oleanolic acid on erythrocyte membrane. Planta Med..

[21] Wokaun A., Ernst R.R. (1977). Selective detection of multiple quantum transitions in NMR by two-dimensional spectroscopy. Chem. Phys. Lett..

[22] Kumar A., Ernst R.R., Wüthrich K. (1980). A Two-dimensional nuclear Overhauser enhancement (2D NOE) Experiment for the elucidation of complete proton-proton cross-relaxation networks in biological macromolecules. Biochem. Biophys. Res. Commun..

[23] Perpickdumont M., Reynolds W.F., Enriquez R.G. (1988). C-13-H-1 shift correlation with full H-1-H-1 decoupling. Magn. Reson. Chem..

[24] Hurd R.E., John B.K. (1991). Gradient-enhanced proton-detected heteronuclear multiple-quantum coherence spectroscopy. J. Magn. Reson..

[25] Mosmann T. (1983). Rapid colorimetric assay for cellular growth and survival: Application to proliferation and cytotoxicity assays. J. Immunol. Methods.

[26] Prayong P., Barusrux S., Weerapreeyakul N. (2008). Cytotoxic activity screening of some indigenous Thai plants. Fitoterapia.

[27] Negera A., Induli M., Fitzpatrick P., Alao J.P., Sunnerhagen P., Landberg G., Yenesew A., Erdélyi M. (2014). Cytotoxic quinones from the roots of *Aloe dawei*. Molecules.

[28] Gupta S., Thapar M.M., Wernsdorfer W.H., Björkman A. (2002). *In vitro* interactions of artemisinin with atovaquone, quinine, and mefloquine against *Plasmodium falciparum*. Antimicrob. Agents Chemother..

[29] Trager W., Jensen J.B. (1976). Human malaria parasites in continuous culture. Science.

[30] Desjardins R.E., Canfield C.J., Haynes J.D., Chulay J.D. (1979). Quantitative assessment of antimalarial activity *in vitro* by a semiautomated microdilution technique. Antimicrob. Agents Chemother..

[31] Batista R., Júnior A.J.S., Oliveira A.B. (2009). Plant-derived antimalarial agents: New leads and efficient phytomedicines. Part II. Non-alkaloidal natural products. Molecules.

