# Supplementary material for: Constituents of the Roots and Leaves of Ekebergia capensis and Their Potential Antiplasmodial and Cytotoxic Activities
Source: Molecules. 2014 Sep 10;19(9):14235–46. doi: 10.3390/molecules190914235 (PMC6270759; doi:10.3390/molecules190914235)
Supplement: Supplementary File 1 [file molecules-19-14235-s001.pdf]

Supplementary Materials

NMR and MS spectra of 3-Oxo-12β-hydroxy-oleanan-28,13β-olide (1)

Figure S1. The ^{1}H-NMR spectrum of compound **1** ($CDCl_3$, 25 C, 799.88 MHz).

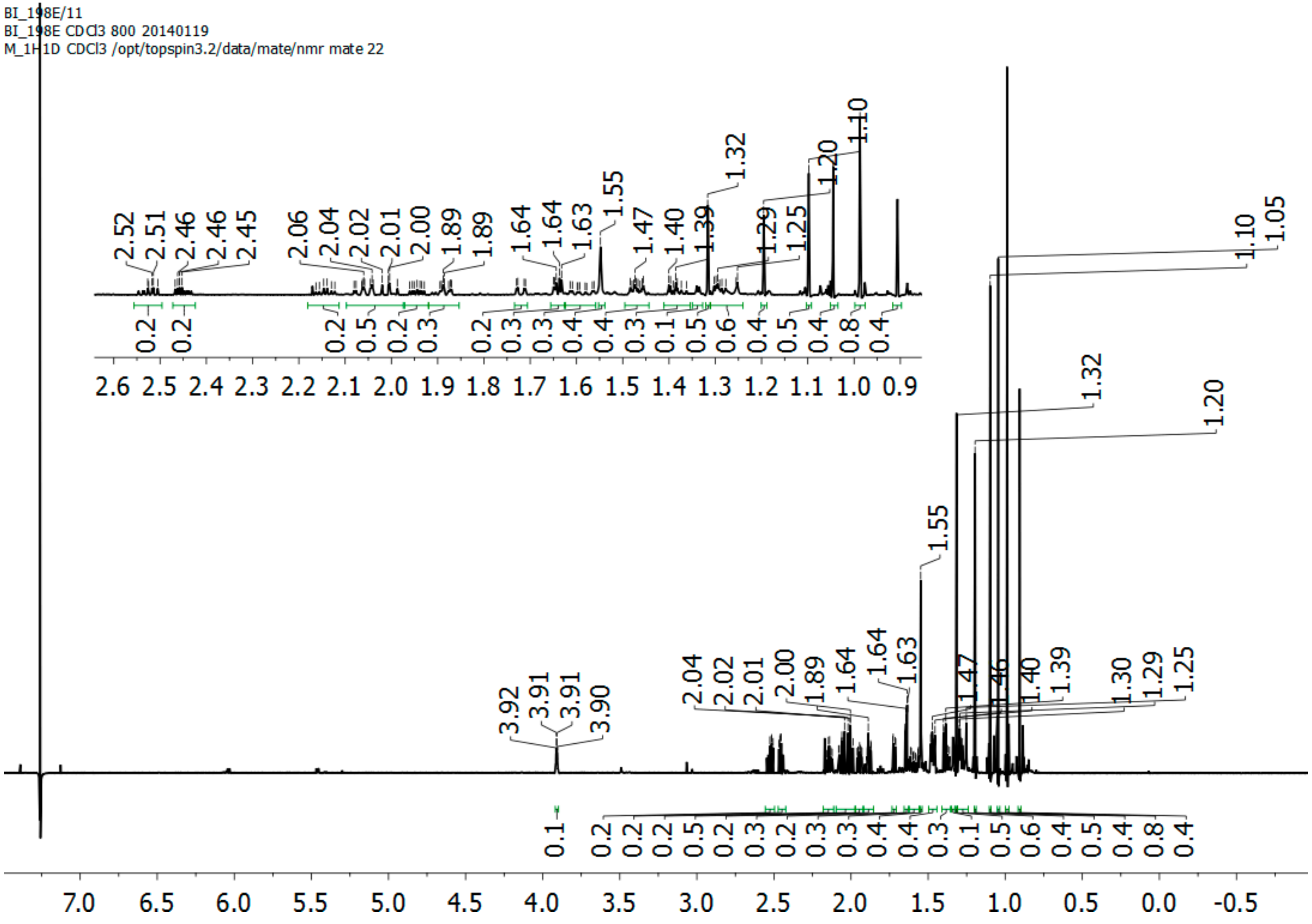

Figure S2. The ^{13}C-NMR spectrum of compound **1** ($CDCl_3$, 25 C, 201.20 MHz).

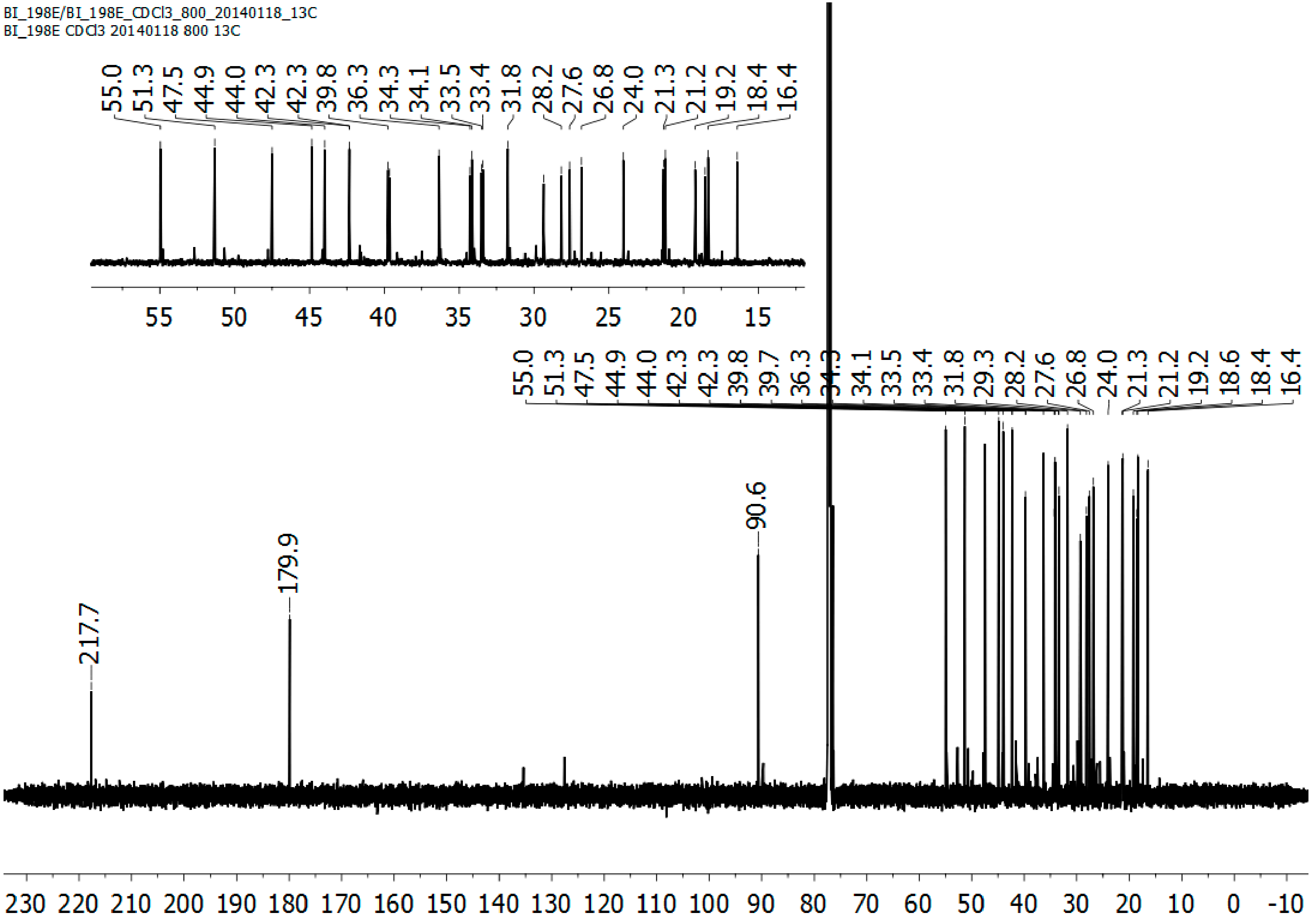

Figure S3. The $^{1}H,^{13}C$ HSQC spectrum of compound **1** ($CDCl_3$, 25 C, 799.88, 201.20 MHz).

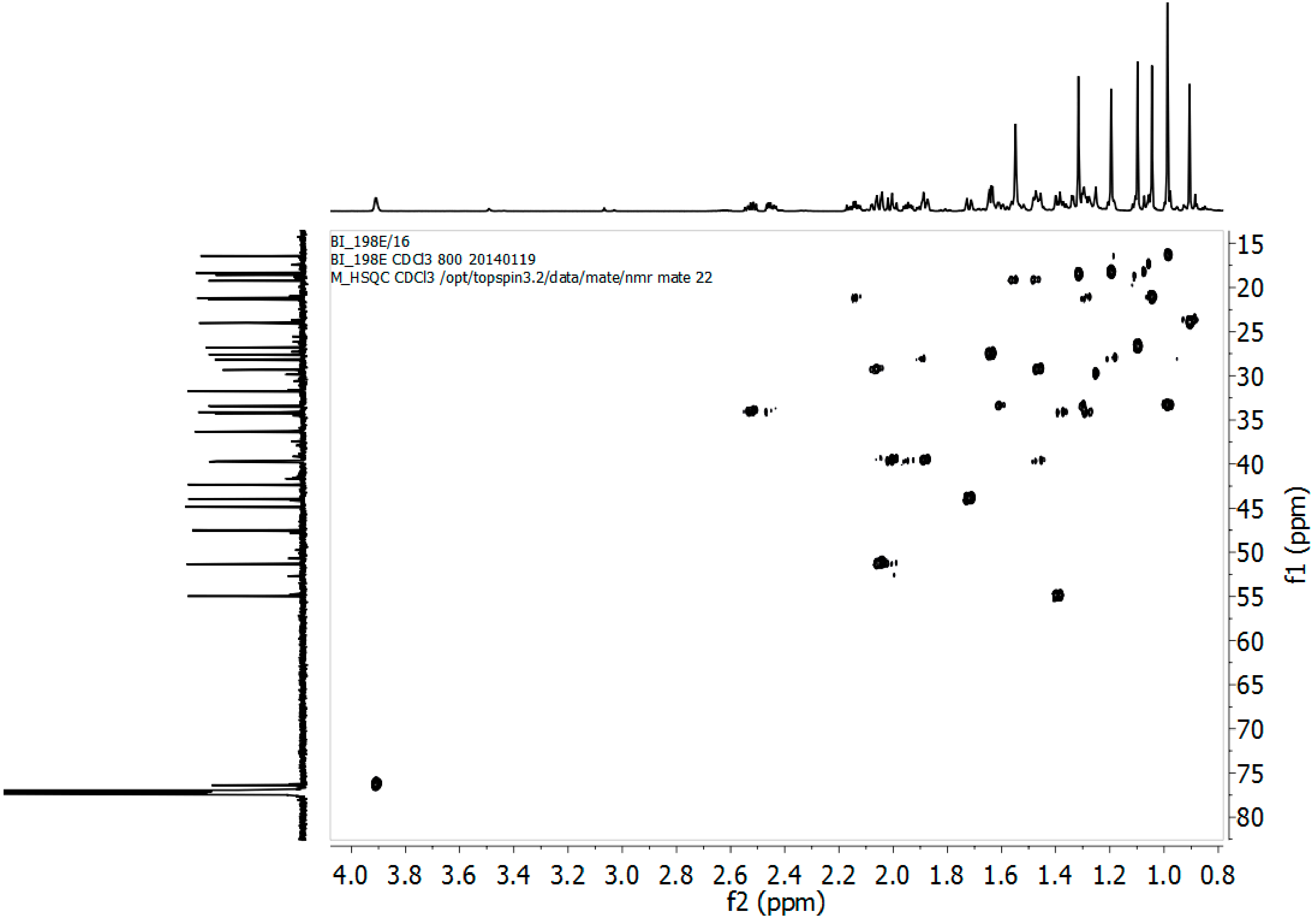

Figure S4. The ^{1}H, ^{13}C HMBC spectrum of compound **1** ($CDCl_3$, 25 C, 799.88, 201.20 MHz).

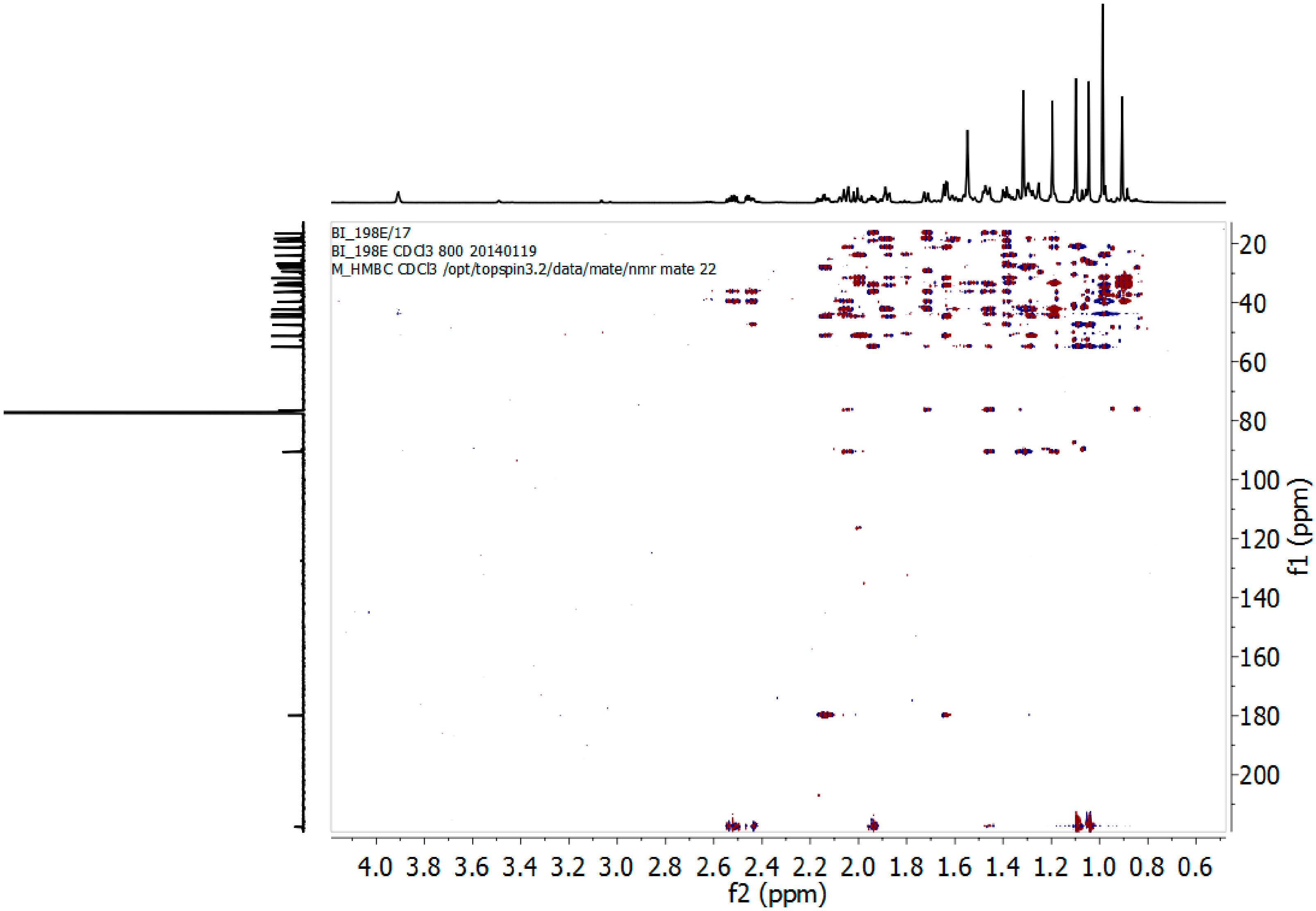

Figure S5. The enlarged aliphatic part of the ^{1}H, ^{13}C HMBC spectrum of compound **1** ($CDCl_3$, 25 C, 799.88, 201.20 MHz).

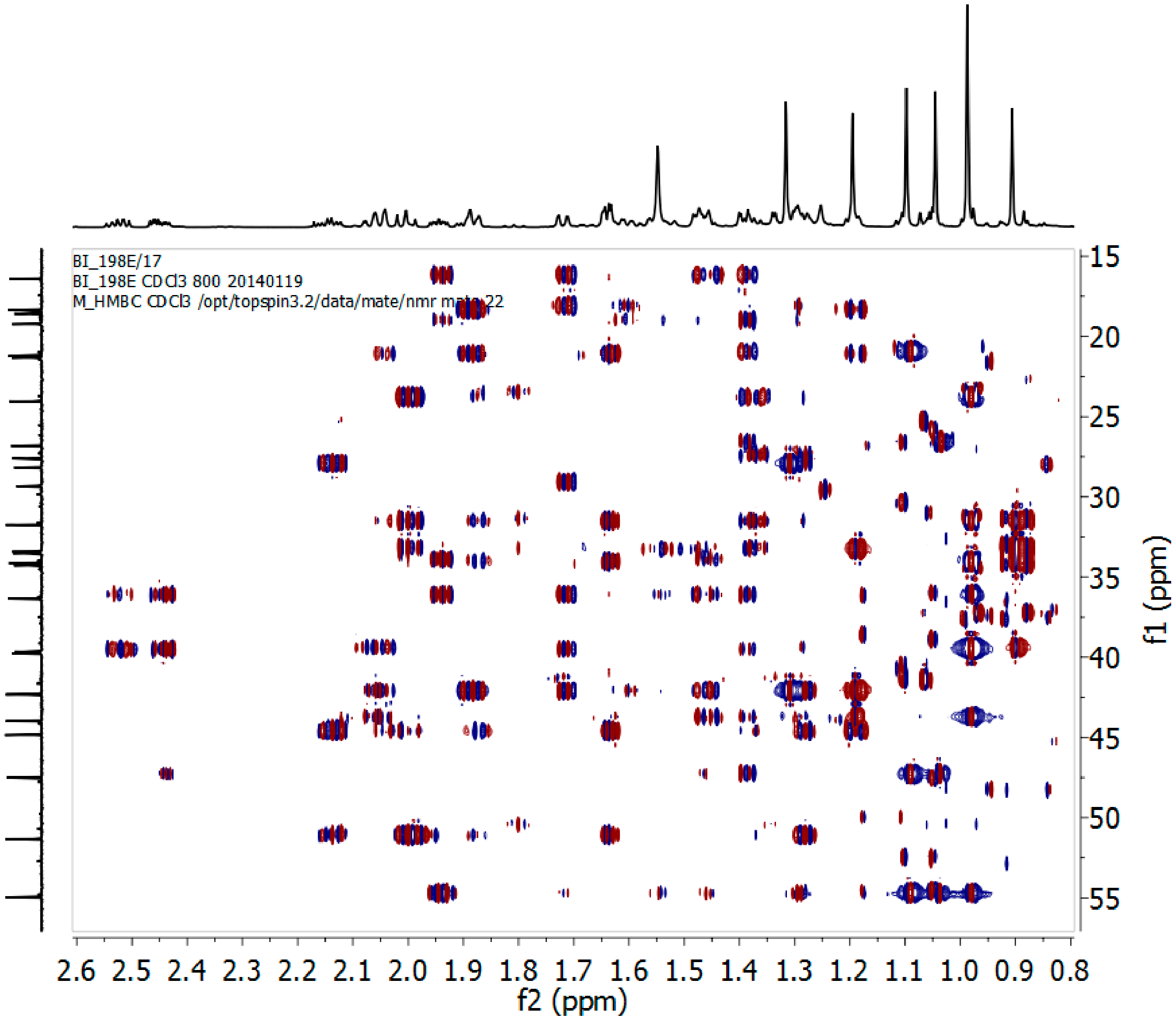

Figure S6. The COSY spectrum of compound **1** ($CDCl_3$, 25 C, 799.88 MHz).

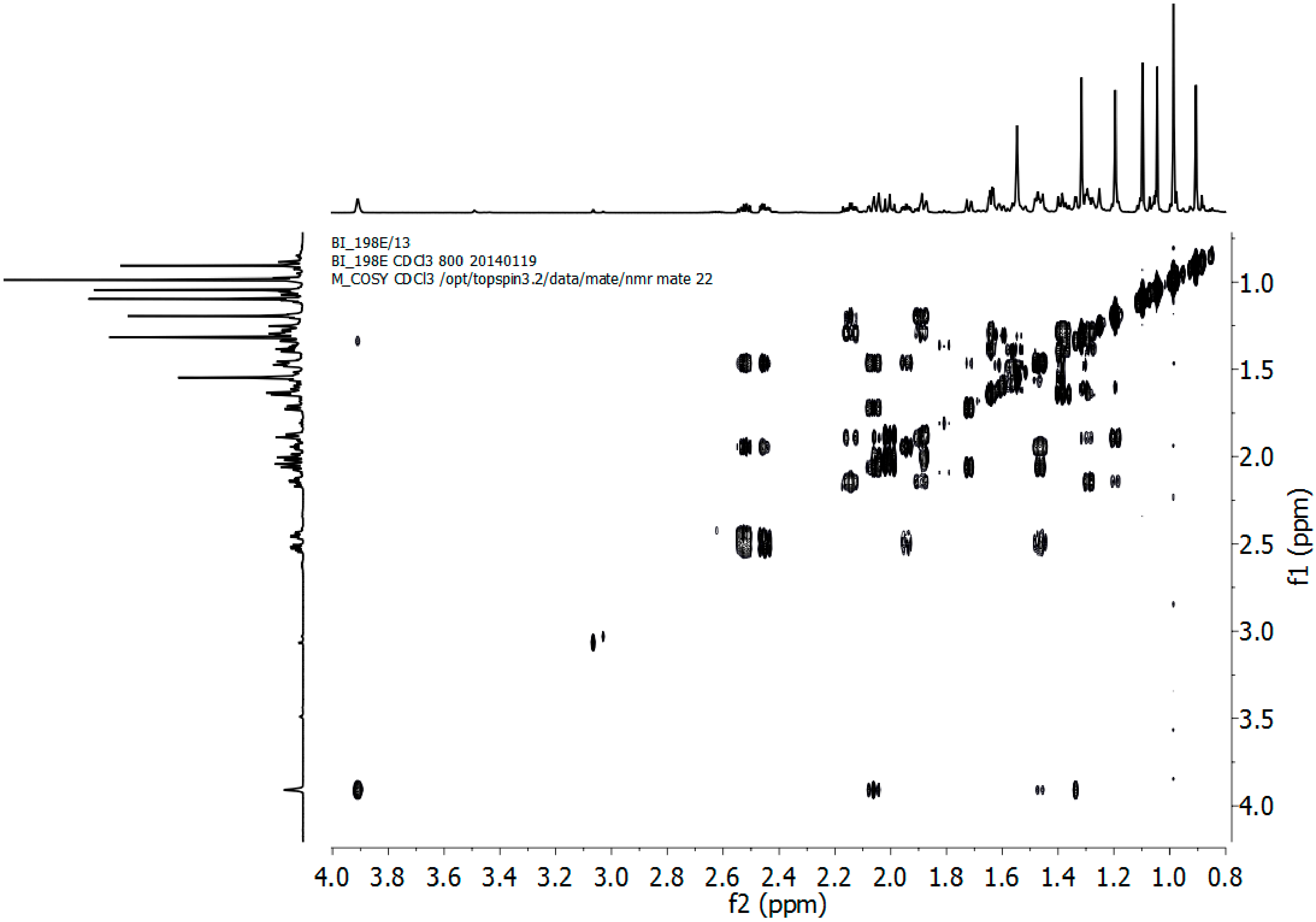

Figure S7. The TOCSY spectrum of compound **1** ($CDCl_3$, 25 C, 799.88 MHz).

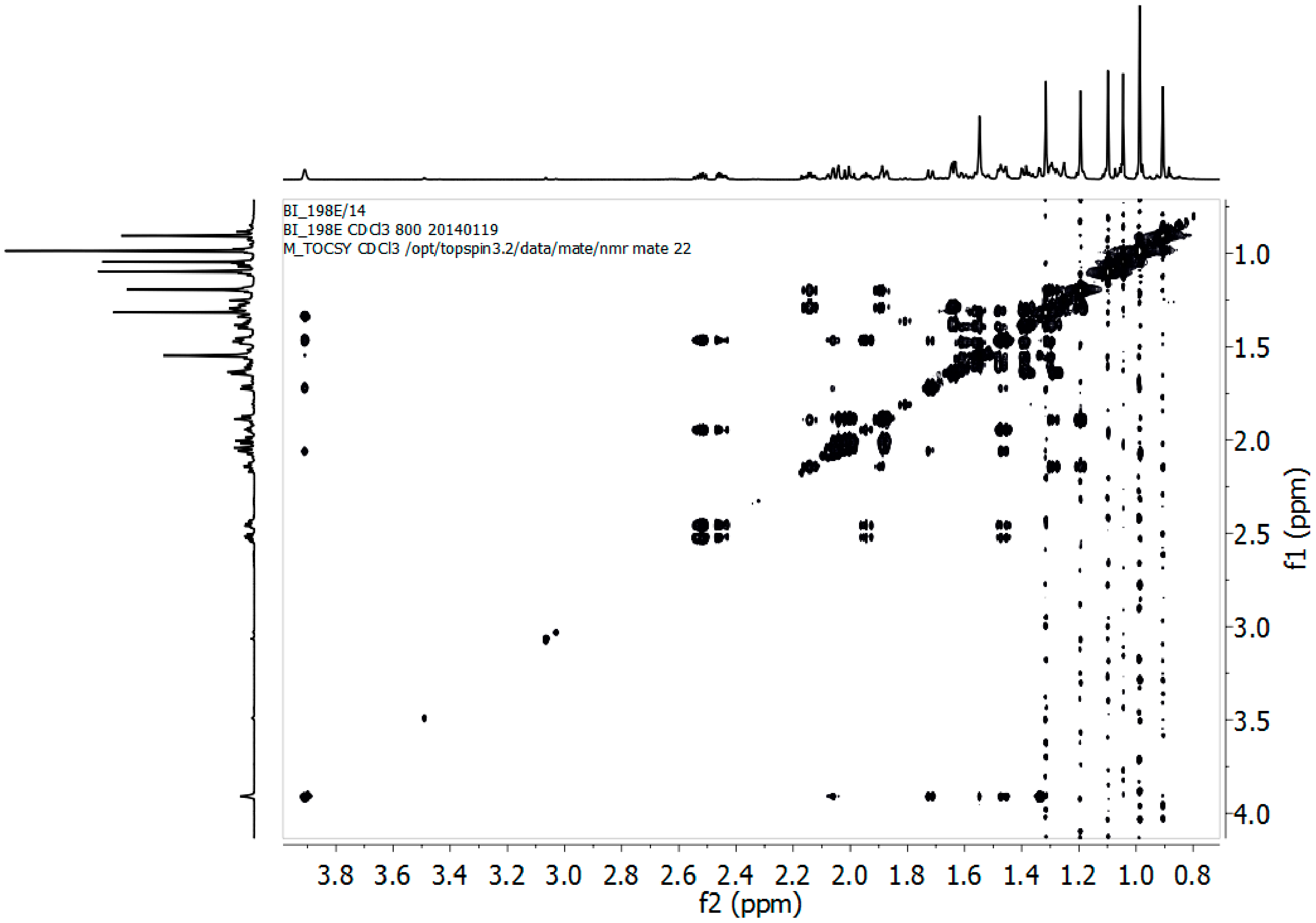

Figure S8. The NOESY spectrum of compound **1** ($CDCl_3$, 25 C, 799.88 MHz).

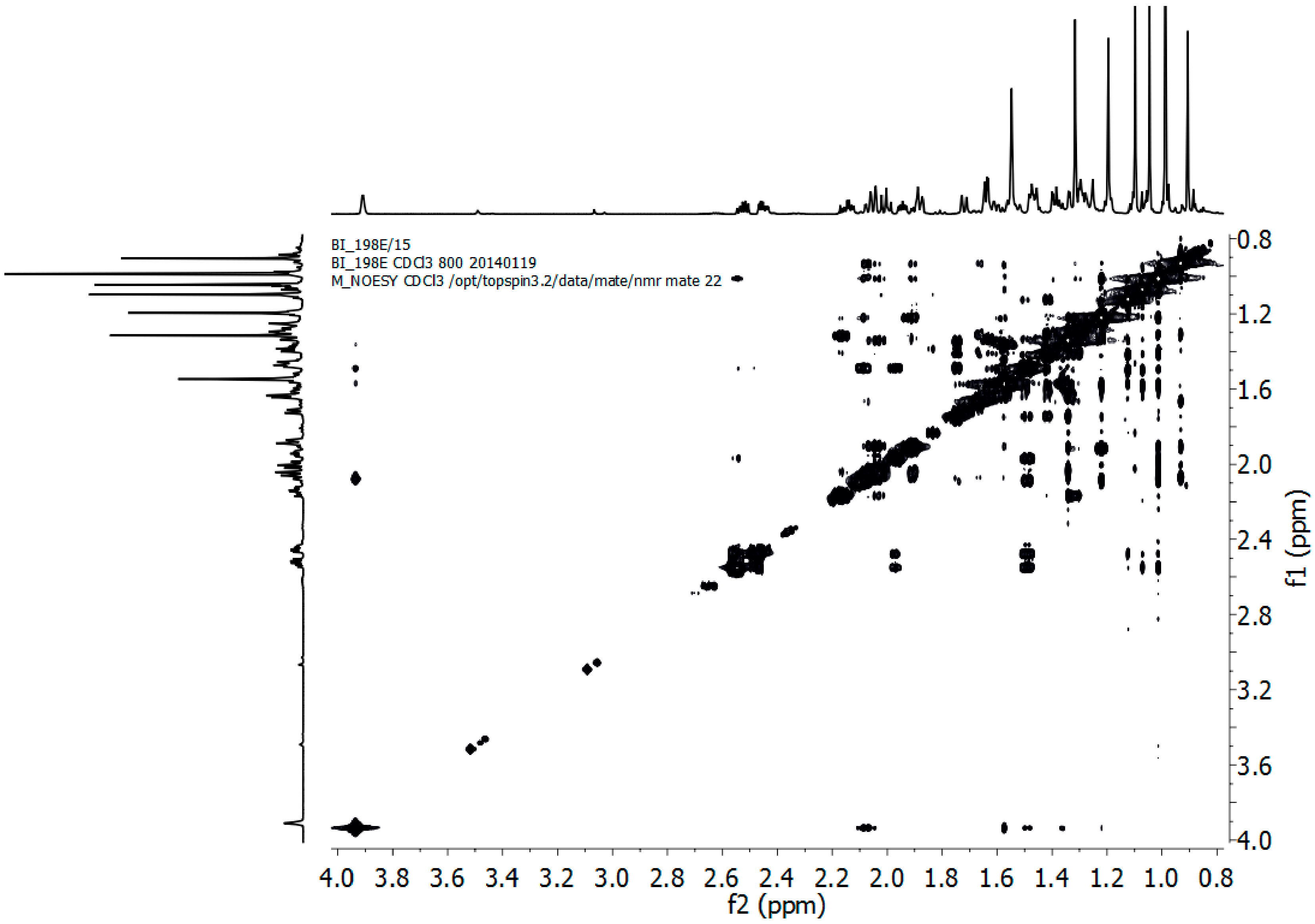

Figure S9. Key COSY correlations observed for compound **1** ($CDCl_3$, 25 °C, 799.88 MHz).

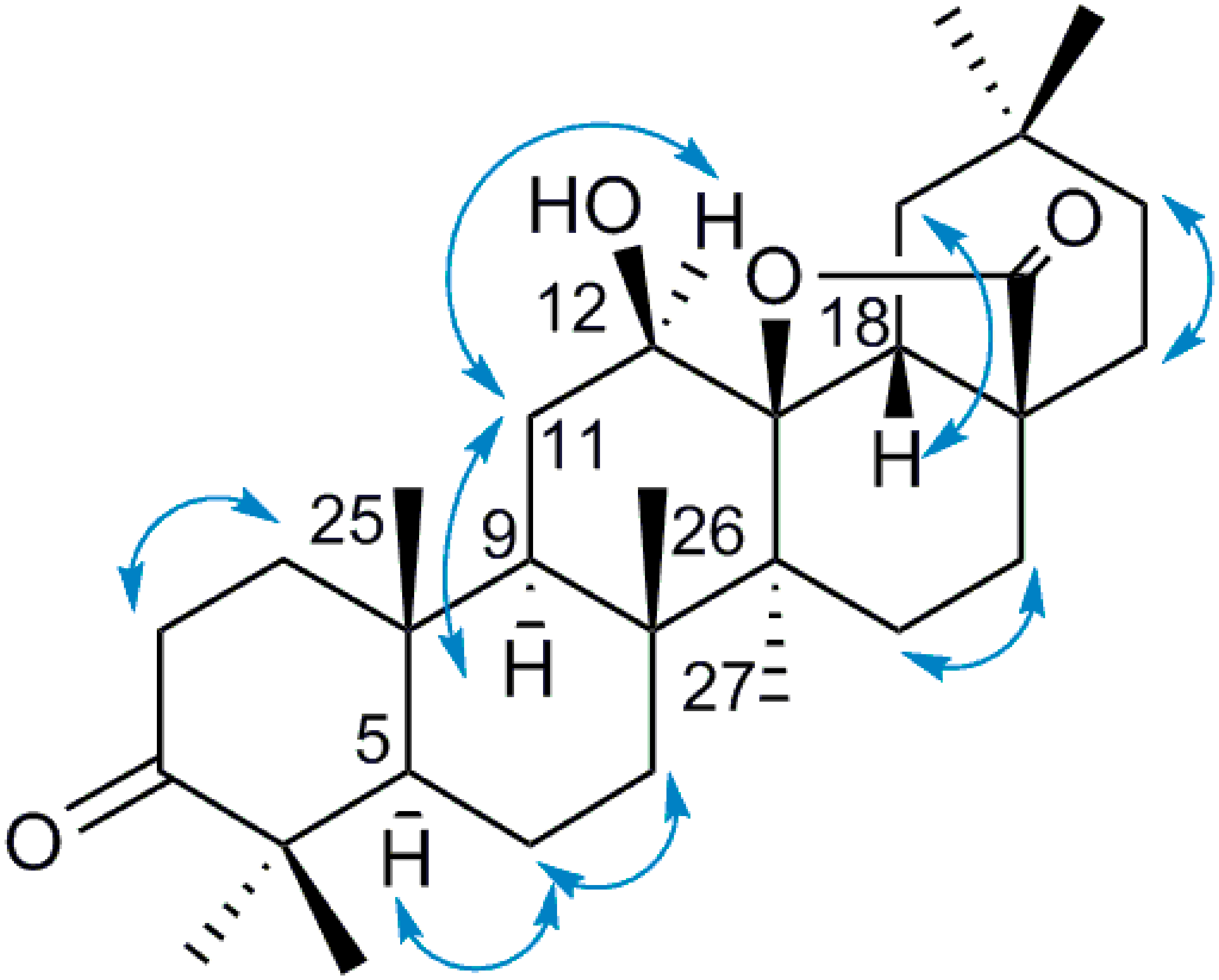

Figure S10. The HR(ESI)MS of compound **1**.

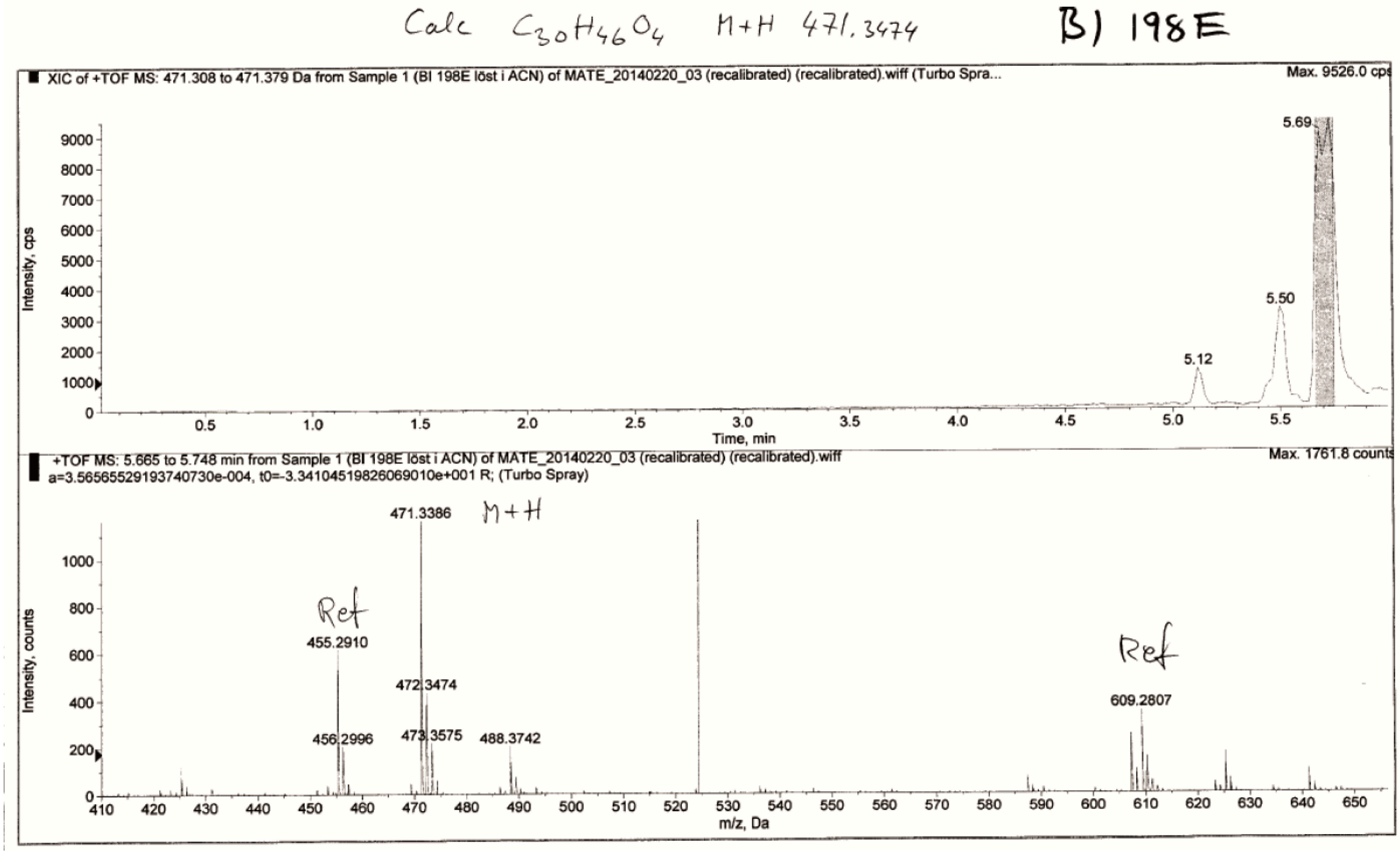

Spectroscopic Data of the Known Compounds 2–10

3-Oxoolean-12-en-28-oic acid (Oleanonic acid) (**2**) [1] ^{1}H-NMR (DMSO-d_6) δ (799.88 MHz, ppm): 0.77 (3H, s, Me-26), 0.88 (6H, Me-29, 30), 0.94 (3H, s, Me-24), 0.97 (3H, s, Me-25), 1.00 (3H, s, Me-23), 1.11 (3H, s, Me-27), 1.38 (1H, m, CH_{2b}-1), 1.77 (1H, ddd, *J* = 11.8, 7.4, 3.8 Hz, CH_{2b}-1), 2.30 (1H, ddd, *J* = 15.9, 7.1, 3.7 Hz, CH_{2a}-2), 2.75 (1H, dd, *J* = 14, 4.5 Hz, CH-18), 5.19 (1H, s, CH-12); ^{13}C-NMR (DMSO-d_6): δ (201.20 MHz, ppm) 15.1 (C-25), 17.2 (C-26), 19.6 (C-6), 21.6 (C-24), 23.1 (C-11), 23.5 (C-16), 23.8 (C-8), 25.9 (C-27), 26.7 (C-23), 27.7 (C-15), 30.9 (C-20), 32.3 (C-7), 32.5 (C-22), 33.3 (C-29), 33.8 (C-21), 34.1 (C-2), 36.7 (C-10), 38.9 (C-1), 29.3 (C-8), 41.4 (C-18), 41.9 (C-14), 46.0 (C-19), 46.1 (C-9), 46.6 (C-17), 47.1 (C-4), 54.8 (C-5), 121.9 (C-12), 144.3 (C-13), 179.1 (C-28), 216.9 (C-3); ESI-MS (30 eV): *m/z* 455.4 $[M+H]^+$.

3-α-Hydroxyolean-12-en-28-oic acid (3-epi-Oleanolic acid) (**3**) [2] ^{1}H-NMR (DMSO-d_6) δ (799.88 MHz, ppm): 0.71 (3H, s, Me-26), 0.75 (3H, s, Me-24), 0.83 (3H, s, Me-23), 0.85 (3H, s, Me-25), 0.86 (6H, s, Me-29), 1.09 (3H, s, Me-27), 2.77(1H, dd, *J* = 15, 5, CH-18), 3.17 (1H, s, CH-3), 5.08 (1H, s, CH-12); ^{13}C-NMR (DMSO-d_6): δ (201.20 MHz, ppm), 15.0 (C-25), 17.0 (C-26), 17.8 (C-6), 22.3 (C-24), 22.9 (C-11), 23.5, (C-16),23.6 (C-30), 25.2 (C-2), 25.7 (C-27), 27.3 (C-15), 28.7 (C-23), 30.5 (C-20), 32.3 (C-1), 32.5 (C-7), 32.6 (C-21), 32.7 (C-22), 33.0 (C-29), 36.7 (C-10), 36.9 (C-4), 41.0 (C-8), 41.4 (C-18), 45.5 (C-14), 46.1 (C-19), 46.9 (C-17), 48.3 (C-9), 48.6 (C-5), 73.8 (C-3), 120.5 (C-12), 144.1 (C-13), 178.3 (C-28).

3-β-Hydroxyolean-12-en-28-oic acid (Oleanolic acid) (**4**) [3] ^{1}H-NMR ($CDCl_3$) δ (500 MHz, ppm): 0.77 (3H, s, Me-26), 0.79 (3H, s, Me-24), 0.92 (3H, s, Me-29), 0.93 (3H, s, Me-25), 0.95 (3H, s, Me-30), 1.00 (3H, s, Me-23), 1.15 (3H, s, Me-27), 2.83 (1H, dd, *J* = 13.8, 4.5 Hz, CH-18), 3.24 (1H, dd, *J* = 10. 5 Hz, CH-3), 5.28 (1H, s, CH-12); ^{13}C-NMR ($CDCl_3$) δ (125.00 MHz, ppm): 15.5 (C-24), 15.3 (C-25), 17.1 (C-26), 18.3 (C-6), 22.9 (C-11), 23.4 (C-16), 23.6 (C-30), 25.9 (C-27), 27.2 (C-2), 28.1 (C-23), 30.7 (C-20), 32.4, (C-22), 32.6 (C-29), 33.1 (C-7), 33.8 (C-21), 37.1 (C-10), 38.4 (C-1), 38.8 (C-4), 39.3 (C-8), 41.0 (C-18), 41.6 (C-14), 45.9 (C-19), 47.6 (C-9), 55.2 (C-5), C-3 (79.0), 122.6 (C-12), 143.6 (C-13), 182.9 (C-28).

Ekeberin A (**5**) [4] ^{1}H-NMR ($CDCl_3$) δ (500 MHz, ppm): 0.88 (3H, s, Me-30), 0.89 (3H, s, Me-29), 0.90 (3H, s, Me-27), 0.96 (3H, s, Me-25), 1.03 (3H, s, Me-24), 1.06 (3H, s, Me-26), 1.07 (3H, s, Me-23), 1.42 (1H, d, *J* = 2.6 Hz, CH-9), 2.03 (1H, ddd, *J* = 13.1, 5.4, 2.5 Hz, CH_{2a}-12), 2.44 (1H, ddd, *J* = 11.3, 7.8, 3.9 Hz, CH_{2a}-2), 2.51 (1H, ddd, *J* = 15.7, 9.5, 7.6 Hz, CH_{2b}-2), 3.44 (1H, dd, *J* = 8.5, 1.7 Hz, CH_{2a}-28), 3.55 (1H, dd, *J* = 10.3, 1,7 Hz, CH-18), 4.25 (1H, dd, *J* = 8.5, 3.2 Hz, CH_{2b}-28); ^{13}C-NMR ($CDCl_3$) δ (125.00 MHz, ppm): 14.8 (C-27), 16.1 (C-26), 16.8 (C-25), 17.4 (C-29), 17.7 (C-30), 20.1 (C-6), 21.5 (C-24), 21.8 (C-11), 24.7 (C-12), 27.2 (C-23), 28.0 (C2-1), 26.9 (C-15), 29.0 (C-16), 31.9 (C-17), 33.3 (C-22), 33.4 (C-7), 34.6 (C-2), 37.5 (C-10), 35.9 (C-20), 39.9 (C-13), 40.3 (C-1), 41.2 (C-8), 41.9 (C-14), 47.8 (C-4), 50.8 (C-9), 55.5 (C-5), 69.6 (C-28), 79.3 (C-18), 98.0 (C-19), 218.6 (C-3).

2-Hydroxymethyl-2,3,22,23-tetrahydroxy-6,10,15,19,23-pentamethyl-6,10,14,18-tetracosatetraene (**6**) [5]: ^{1}H-NMR (DMSO-d_6) δ (799.88 MHz, ppm): 0.93 (3H, s, Me-25), 0.96 (3H, s, Me-24), 0.99 (3H, s, Me-30), 1.50 (12H, s, Me-26, 27, 28, 29), 3.03 (1H, d, *J* = 10.2 Hz, CH-22), 3.20 (1H, d, *J* = 10.8 Hz,

CH_{2a}-1), 3.24 (1H, d, J = 10.4 Hz, CH-3), 3.32 (1H, d, J = 10.8 Hz, CH_{2b}-1), 5.04 (4H, br s, CH-7, 11, 14, 18); ^{13}C-NMR (DMSO-d_6); δ (201.20 MHz, ppm): 16.33, 16.34 16.4, 16.5, (Me-26, 27, 28, 29), 21.22 (C-25), 25.2 (C-24), 26.0 (C-30), 26.65, 26.7 (C-17 and C-8), 28.3 (C-11/C-12), 29.8 (C-4), 30.1 (C-21), 36.9 (C-5), 37.1 (C-20), 39.82 (C-9), 39.83 (C-16), 67.2 (C-1), 72.4 (C-23), 74.5 (C-2), 74.6 (C-3), 124.0 (C-11/C-14), 124.41 (C-7), 124.44 (C-18), 134.82 (C-6), 134.87 (C-10/C-15), 134.93 (C10/C15), 134.97 (C19); ESI-MS (30 eV): *m/z* 495.7 $[M+H]^+$.

2,3,22,23-Tetrahydroxy-2,6,10,15,19,23-hexamethyl-6,10,14,18-tetracosate (**7**) [5] ^{1}H-NMR ($CDCl_3$) δ (500 MHz, ppm): 1.13 (6H, s, Me-1, 24), 1.18 (3H, s, Me-25, 30), 1.58 (6H, Me-27, Me-28), 1.60 (6H, s, Me-26, 29), 3.33 (2H, dd J = 10.5, 1.9 Hz), 5.13 (2H, m, CH-11, 14) 5.17 (1H, t, J = 6.4 Hz, CH-7); ^{13}C-NMR ($CDCl_3$) δ (125.00 MHz, ppm): 16.3 (C-27/C-28), 16.4 (C-26/C-29), 23.6 (C-1/C-24), 26.78 (C-8), 26.8 (C-17), 26.9 (C-25), 28.6 (C-12/C-13), 30.1 (C-4/C-21), 37.2 (C-5/C-20), 40.0 (C-9/C-16), 73.4 (C-2/C-23), 78.6 (C-3/C-22), 124.8 (C-11/C-14), 125.4 (C-7/C-18), 135.2 (6, 10, 15, 19), 135.3(6, 10, 15,19); ESI-MS (30 eV): *m/z* 479.4 $[M+H]^+$.

Proceranolide (**8**) [6] ^{1}H-NMR ($CDCl_3$) δ (799.88 MHz, ppm): 0.73 (3H, s, Me-29), 0.81 (3H, s, Me-28), 1.03 (3H, s, Me-18), 1.12 (3H, s, Me-19), 2.38 (1H, m, CH_{2a}-6), 2.38 (1H, m, CH_{2b}-6), 3.05 (1H, ddd, J = 10.7, 6.0, 2.7, Hz, CH-2), 3.19 (1H, dd, J = 14.5, 3.0 Hz, CH-30), 3.24 (1H, dd, J = 11.4, 2.7 Hz, CH-5), 3.74 (1H, m, CH-3), 3.70 (3H, s, OMe), 4.05 (1H, dt, J = 21.4, 2.12, 2.12 Hz, CH-15), 5.58 (1H, s, CH-17), 6.49 (1H, d, J = 2.3 Hz, CH-22), 7.39 (1H, s, CH-23), 7.56 (1H, s, CH-21); ^{13}C-NMR ($CDCl_3$); δ (201.20 MHz, ppm): 17.2 (C-19), 19.1(C-11), 20.5 (C-29), 24.1 (C-28), 28.9 (C-12), 33.4 (C-15), 33.6 (C-6), 33.9 (C-30), 38.2 (C-13), 39.6 (C-5), 50.3 (C-2), 52.1 (OMe), 52.3 (C-9), 53.9 (C-10), 77.5 (C-3), 80.5 (C-17), 110.4 (C-22), 121.1 (C-20), 128.5 (C-8), 131.7 (C-14), 142.0 (C-21), 142.9 (C-23), 171.7 (C-16), 174.7 (C-7), C-1 (207.3); ESI-MS (30 eV): *m/z* 471.9 $[M+H]^+$.

Kaempferol-3-O-β-D-glucopyranoside (**9**) [7] ^{1}H-NMR (DMSO-d_6) δ (799.88 MHz, ppm): 3.08 (2H, s, CH-4'', CH-5''), 3.13 (1H, m, CH_{2a}-6''), 3.17 (1H, s, CH-2''), 3.21 (1H, s, CH-3''), 3.56 (1H, m, CH_{2b}-6''), 5.46 (1H, d, J = 7.2 Hz, CH-1''), 6.20 (1H, s, CH-6), 6.42 (1H, s, CH-8), 6.88 (2H, m, CH-3', CH-5') 8.04 (2H, d, J = 7.2 Hz, CH-2', CH-6'); ^{13}C-NMR (DMSO-d_6) δ (201.20 MHz, ppm): 61.3 (C-6''), 70.4 (C-4''), 74.7 (C-2''), 76.9 (C-3''), 78.0 (C-5''), 94.1 (C-8), 99.2 (C-6), 101.3 (C-1''), 104.4 (C-10), 115.6 (C-3'/C-5'), 121.4 (C-1'), 131.3 (2'/6'), 133.6 (C-3), 156.1 (C-2), 156.9 (C-9), 160.4 (4'), 161.9 (C-5), 177.9 (C-4); ESI-MS (30 eV): *m/z* 449.1 $[M+H]^+$.

Quercetin-3-O-β-D-glucopyranoside (**10**) [8] ^{1}H-NMR (DMSO-$d_{6)}$ δ (799.88 MHz, ppm): 3.08 (2H, s, CH-4''/ CH-5''), 3.22 (2H, 2, CH-2''/ CH-3''), 3.33 (1H, m, CH_{2a}-6''), 3.58 (1H, dd, J = 11.9, 4 Hz, CH_{2b}-6''), 5.46 (1H, d, J = 7.4 Hz, CH-1''), 6.20 (1H, d, J = 2.0 Hz, CH-6), 6.40 (1H, d, J = 2.0 Hz, CH-8), 6.84 (1H, d, J = 10.0 Hz, CH-5'), 7.57 (1H, m, CH-2'), 7.58 (1H, m, CH-6'); ^{13}C-NMR (DMSO-d_6); δ (201.20 MHz, ppm): 61.0 (C-6''), 69.9 (C-4''), 74.1 (C-2''), 76.5 (C-3''), 77.6 (C-5''), 93.5 (C-8), 98.6 (C-6), 100.8 (C-1''), 104.0 (C-10), 115.2 (C-5'), 116.2 (C-2'), 121.2 (C-1'), 121.6 (C-6'), 133.3 (C-3), 144.8 (3'), 148.5 (C-4'), 156.2 (C-2), 156.3 (C-9), 161.2 (C-5), 164.1 (C-7), 177.4 (C-4); ESI-MS (30 eV): *m/z* 465.1 $[M+H]^+$.

References

1. Kwon, H.C.; Lee, K.R.; Zee, O.P. Cytotoxic constituents of *Pilea mongolica*. *Arch. Pharm. Res.* **1997**, *20*, 180–183.
2. Sewram, V.; Raynor, M.W.; Mulholland, D.A.; Raidoo, D.M. The uterotonic activity of compounds isolated from the supercritical fluid extract of *Ekebergia capensis*. *J. Pharm. Biomed. Anal.* **2000**, *24*, 133–145.
3. Seebacher, W.; Simic, N.; Weis, R.; Saf, R.; Kunert, O. Complete assignments of ^{1}H and ^{13}C-NMR resonances of oleanolic acid, 18α-oleanolic acid, ursolic acid and their 11-oxo derivatives. *Magn. Reson. Chem.* **2003**, *41*, 636–638.
4. Murata, T.; Miyase, T.; Muregi, F.W.; Naoshima-Ishibashi, Y.; Umehara, K.; Warashina, T.; Kanou, S.; Mkoji, G.M.; Terada, M.; Ishih, A. Antiplasmodial Triterpenoids from *Ekebergia capensis*. *J. Nat. Prod.* **2008**, *71*, 167–174.
5. Nishiyama, Y.; Moriyasu, M.; Ichimaru, M.; Tachibana, Y.; Kato, A.; Mathenge, S.G.; Nganga, J.N.; Juma, F.D. Acyclic triterpenoids from *Ekebergia capensis*. *Phytochemistry* **1996**, *42*, 803–807.
6. Kadota, S.; Marpaung, L.; Kikuchi, T.; Ekimoto, H. Constituents of the seeds of Swietenia mahagoni Jacq. II. Structures of swietemahonin A, B, C, D, E, F, and G and swietemahonolide. *Chem. Pharm. Bull.* **1990**, *38*, 894–901.
7. Tang, R.; Chen, K.; Cosentino, M.; Lee, K.-H. Apigenin-7-*O*-β-D-glucopyranoside, an anti-HIV principle from *Kummerowia striata*. *Bioorg. Med. Chem. Lett.* **1994**, *4*, 455–458.
8. Kim, H.Y.; Moon, B.H.; Lee, H.J.; Choi, D.H. Flavonol glycosides from the leaves of *Eucommia ulmoides* O. with glycation inhibitory activity. *J. Ethnopharmacol.* **2004**, *93*, 227–230.
